# Silver Nanoparticles Effects on In Vitro Germination, Growth, and Biochemical Activity of Tomato, Radish, and Kale Seedlings

**DOI:** 10.3390/ma14185340

**Published:** 2021-09-16

**Authors:** Alicja Tymoszuk

**Affiliations:** Laboratory of Ornamental Plants and Vegetable Crops, Faculty of Agriculture and Biotechnology, Bydgoszcz University of Science and Technology, Bernardyńska 6, 85-029 Bydgoszcz, Poland; alicja.tymoszuk@pbs.edu.pl; Tel.: +48-52-374-95-64

**Keywords:** carotenoids, chlorophylls, enzymatic activity, nanotechnology, micropropagation, polyphenols, vegetable crops

## Abstract

The interactions between nanoparticles and plant cells are still not sufficiently understood, and studies related to this subject are of scientific and practical importance. Silver nanoparticles (AgNPs) are one of the most commonly produced and used nanomaterials. This study aimed to investigate the influence of AgNPs applied at the concentrations of 0, 50, and 100 mg·L^−1^ during the process of in vitro germination as well as the biometric and biochemical parameters of developed seedlings in three vegetable species: *Solanum lycopersicum* L. ‘Poranek’, *Raphanus sativus* L. var. *sativus* ‘Ramona’, and *Brassica oleracea* var. *sabellica* ‘Nero di Toscana’. The application of AgNPs did not affect the germination efficiency; however, diverse results were reported for the growth and biochemical activity of the seedlings, depending on the species tested and the AgNPs concentration. Tomato seedlings treated with nanoparticles, particularly at 100 mg·L^−1^, had shorter shoots with lower fresh and dry weights and produced roots with lower fresh weight. Simultaneously, at the biochemical level, a decrease in the content of chlorophylls and carotenoids and an increase in the anthocyanins content and guaiacol peroxidase (GPOX) activity were reported. AgNPs-treated radish plants had shorter shoots of higher fresh and dry weight and longer roots with lower fresh weight. Treatment with 50 mg·L^−1^ and 100 mg·L^−1^ resulted in the highest and lowest accumulation of chlorophylls and carotenoids in the leaves, respectively; however, seedlings treated with 100 mg·L^−1^ produced less anthocyanins and polyphenols and exhibited lower GPOX activity. In kale, AgNPs-derived seedlings had a lower content of chlorophylls, carotenoids, and anthocyanins but higher GPOX activity of and were characterized by higher fresh and dry shoot weights and higher heterogeneous biometric parameters of the roots. The results of these experiments may be of great significance for broadening the scope of knowledge on the influence of AgNPs on plant cells and the micropropagation of the vegetable species. Future studies should be aimed at testing lower or even higher concentrations of AgNPs and other NPs and to evaluate the genetic stability of NPs-treated vegetable crops and their yielding efficiency.

## 1. Introduction

Engineered nanomaterials (ENMs) are atomic or molecular aggregates, which, due to their small size between 1 and 100 nm, present unique and different chemical and physical characteristics from those at the micrometric scale, such as a large surface-area-to-volume ratio, the ability to engineer electron exchange, high surface reactive capabilities, high reactivity, and easy absorption by cells. Over the past few decades, ENMs have been used in industrial sectors and in daily life, in biotechnology, and in agriculture, which has led to their inevitable release into the environment and ecosystems and has created possible hazards for all living organisms, including plants. Understanding the influence of ENMs on plants is crucial, not only from the scientific point of view but also for the assessment of environmental risks on food safety and human health [1,2,3,4,5].

A variety of nanomaterials has been developed, including carbon nanotubes, iron, aluminum, copper, gold, silver, silica, zinc, zinc oxide, titanium oxide, among others [6]. Silver nanoparticles (AgNPs) are the most commonly used nanomaterial; nearly 25% of all nanotechnology products involve AgNPs. The antimicrobial and antifungal properties have contributed to their widespread use in healthcare, medical devices, building materials, textiles, cosmetics, household products, and foodservice. The good electrical conductivity and photochemical properties result in their implementation in electronic devices and wastewater treatment. Silver nanoparticles are also used in agriculture as plant growth stimulators, fungicides, or agents to enhance fruit ripening [4,5]. AgNPs are assumed to modify the structural components of cellular membranes, macromolecules, influence cell division and defense systems, and interfere in the physiological and biochemical processes of plants by altering gene expression [7].

The influence of ENMs on living organisms is complex, and interestingly, the responses of plants to nanomaterials are often contradictory. The same type of nanomaterial may have various biological impacts depending on the plant genotype, organ, age, and developmental stage; physicochemical properties, concentration, size, shape, surface area, surface coatings, and the stability of the particles or solvent applied as well as the experimental methodology (application to medium, soil, hydroponics; exposure time or method) [1,5,8]. Plant tissue cultures provide an ideal environment to study the effect of ENMs on plants. The sterile environment and the chemically defined culture medium composition avoid most of the extraneous factors that could influence nanoparticle interaction with plants. The positive aspects of different ENMs applications in plant in vitro cultures have been reported and are most often for the elimination of microbial contamination, the induction of caulogenesis, adventitious organogenesis, somatic embryogenesis, genetic transformation, metabolite production [9], and for improving the efficiency of cryopreservation protocols [10]. Nevertheless, nanomaterials may also negatively influence plant metabolism, growth, and development. They are also used as mutagenic factors to induce genetic and phenotypic variation that is valuable in breeding [11,12]. The phytotoxicity of ENMs may be associated with the generation of reactive oxygen species (ROS), leading to lipid peroxidation and disturbances in the redox state of cells and, consequently, oxidative stress [6,13]. Superoxide dismutase (SOD), catalase (CAT), ascorbate, or guaiacol peroxidase (APX, GPOX) are enzymatic antioxidants that catalyze the decomposition of ROS, and changes in their activity are considered as biological markers of oxidative stress [10,14]. Moreover, flavonoids (including anthocyanins) and polyphenols are antioxidant molecules that are produced by plants and are mainly for protection against stresses and are involved in detoxification reactions, acting as metal chelators and participating in ROS scavenging through peroxidases [13,15]. The content of chlorophyll as a major component of chloroplasts influences photosynthesis efficiency. However, stress conditions may decline the content of this pigment, which directly relates to a reduction in the photosynthetic rate and, consequently, in plant growth and development inhibition [7].

Seed germination, beginning with dry mature seed imbibition and ending with a radical protrusion, is the first step of plant growth and is not only crucial for seedling establishment but is also important for crop yield [16]. The germination process is the most sensitive stage of higher plant ontogenesis. Various internal and external factors can modulate catabolic and anabolic processes during germination, thus studying the effects of ENMs during this phase seems to be very informative for researchers and agronomists, especially when looking at edible plants [1,17]. The assessment of nanoparticles toxicity on plants usually involves the measurement of germination time and rate, root elongation, shoot, and root biomass or root tip morphology [8]. Several experiments have been conducted with the use of different ENMs, e.g., gold, silver, copper, silicon, zinc oxide, titanium oxide nanoparticles, carbon structures (fullerenes, nanotubes, graphene), quantum dots, showing diverse (stimulating or phytotoxic), dose- and species-dependent effects on germination efficiency, as well as further seedling growth and development [17,18]. The application of zinc oxide nanoparticles at a wide range of concentrations from 50 to 1600 mg·L^−1^ positively affected the germination in *Allium cepa* L. with no negative effects on the further growth and development of seedlings being observed [19]. Interestingly, Fe_2_O_3_ nanocubes, Fe_2_O_3_ short nanorods, Fe_2_O_3_ long nanorods, and TiO_2_ NPs tested at concentrations 5–150 mg·L^−1^ inhibited the germination of rice (*Oryza sativa* L.) seeds but promoted the growth of roots and shoots and had no effect on the fresh weight [20]. Conversely, AgNPs increased the efficiency of seed germination but inhibited seedling growth in *Pennisetum glaucum* L. (50 mg·L^−1^) [21]; decreased the share of germinating seeds in *Alnus subcordata* L. (10 and 20 mg·L^−1^) [22]; and had no effect on germination and reduced root length in *Zea mays* L. (0.005–2.5 mg·L^−1^) [23].

Despite the fact that different studies have been performed on the regulation of seed germination, the interactions between ENMs and plant cells are not yet fully understood, and require further investigation and elucidation, especially due to the hazards of using NPs treatments in plants. Therefore, the present research aimed to broaden the scope of knowledge on the influence of AgNPs applied at the concentrations of 50 and 100 mg·L^−1^ on the in vitro seed germination, growth, and biochemical activity of developed seedlings in three vegetable species that are known and commonly consumed worldwide—*Solanum lycopersicum* L. (Solanaceae), *Raphanus sativus* L. var. *sativus* (Brassicaceae), and *Brassica oleracea* var. *sabellica* L. (Brassicaceae). The simultaneous testing of species belonging to the same or different botanical family and differing in terms of traits such as seed size, germination dynamics and growth of vegetative organs, and the type of organ used as a crop allows the multifaceted influence of silver nanoparticles on plants to a high extent to be determined.

## 2. Materials and Methods

### 2.1. In Vitro Culture Medium

The modified Murashige and Skoog (MS) basal medium was used [24] with the content of calcium and iron (660 mg·L^−1^ CaCl_2_·2H_2_O, 41.7 mg·L^−1^ FeSO_4_·7H_2_O; 55.8 mg·L^−1^ Na_2_EDTA·2H_2_O) (Chemia, Bydgoszcz, Poland) increased by half. The medium contained 30 g·L^−1^ sucrose and was solidified with 0.8% (*w*/*v*) Plant Propagation LAB-AGAR^TM^ (BIOCORP, Warsaw, Poland). No plant growth regulators (PGR) were added. The medium pH was set to 5.8 after adding all of the nutrients. Next, 40 mL of the medium was poured into 350-mL glass jars sealed with plastic and was autoclaved at 121 °C for 20 min.

### 2.2. In Vitro Physical Growing Conditions

In vitro cultures were kept in the growth room at a temperature of 23 ± 1 °C with a 16/8-h day/night regime. Philips TLD 36W/54 fluorescent lamps emitting cool daylight (Koninklijke Philips Electronics N.V., Eindhoven, The Netherlands) were used as the light source. The photosynthetic photon flux density was set at 35 μmol·m^−2^·s^−1^.

### 2.3. Characteristics of Nanoparticles

**Figure 1:**
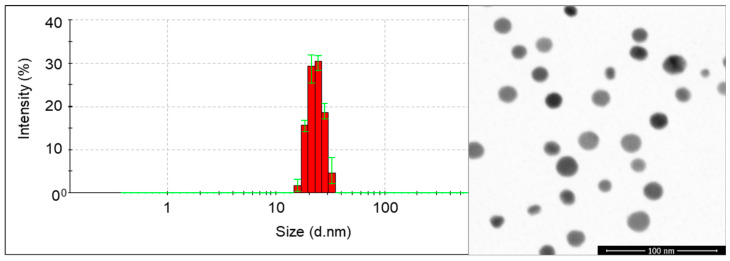
The DLS size distribution graph and STEM image (from the left) of the tested AgNPs (figures provided by courtesy the of Nanoparticles Innovation NPIN s.c., Łódź, Poland).

In the experiments, silver nanoparticles (AgNPs) manufactured by Nanoparticles Innovation NPIN s.c. (Łódź, Poland) were used. The AgNPs were produced by the seeded-mediated growth method [25,26]. The hydrodynamic size of the AgNPs in colloids was 23 ± 4 nm, confirmed by Dynamic Light Scattering (DLS; Nano ZS Zetasizer system, Malvern Instruments, Malvern, UK). The size and size distribution were measured by Scanning Transmission Electron Microscopy (STEM) (Nova NanoSEM 450, FEI™, Hillsboro, OR, USA) at an accelerating voltage of 30 kV and reached 20 ± 3 nm (Figure 1).

### 2.4. Plant Material—Tested Genotypes and In Vitro Culture Initiation

In the experiments, seeds of tomato *Solanum lycopersicum* L. ‘Poranek’, radish *Raphanus sativus* L. var. *sativus* ‘Ramona’, and kale *Brassica oleracea* var. *sabellica* L. ‘Nero di Toscana’ were used. Seeds were purchased at PlantiCo—Hodowla i Nasiennictwo Ogrodnicze Zielonki Sp. z o.o. (Zielonki Parcela, Poland). Before establishing the in vitro culture, the seeds were surface disinfected. First, they were rinsed in running tap water. Next, the seeds were incubated in sequence in a 5% (*v/v*) detergent solution for 5 min; a 70% (*v/v*) ethanol solution for 1 min; and a 1.5% (*v/v*) NaClO solution for 10 min (all chemicals provided by Chemia, Bydgoszcz, Poland). Afterward, the seeds were rinsed twice in sterile bi-distilled water for 5 min. Then, the seeds were inoculated on the modified MS medium, with five seeds in each jar. At concentrations of 50 and 100 mg·L^−1^, the colloid of the AgNPs was poured with a sterile pipette tip and was gently mixed to cover the entirety of the medium and seed surfaces (1 mL per each culture jar). Seeds poured with sterile bi-distilled water were the control object. For each experimental treatment, 20 repetitions were used with 5 seeds in each (1 jar). During the first week of the experiment, the jars were covered with aluminum foil to limit access to light.

### 2.5. Plant Material—Observations of In Vitro Cultures and Biometric Evaluation

Observations of seed germination and seedling growth were performed weekly for three successive weeks. The dynamics of seed germination were determined based on the number of germinating seeds. The biometric data of all of the developed three-week-old seedlings, such as the shoot length (cm), fresh weight of the shoot (mg), dry weight of the shoot (mg), the root length (cm), fresh weight of the root (mg), and dry weight of the root (mg), were collected.

### 2.6. Plant Material—Biochemical Array

The leaves of three-week-old seedlings were used as fresh tissue samples. Chlorophylls and carotenoids were extracted according to Lichtenthaler [27] using 100% acetone (Chemia, Bydgoszcz, Poland) and 100 mg samples. The analysis of the total phenolic content was performed based on the Folin–Ciocalteau procedure [28] and used 200 mg samples with gallic acid (Sigma-Aldrich, St. Louis, MO, USA) as the calibration standard. For the determination of enzymatic activity, 100 mg samples were homogenized in 100 mM phosphate buffer (pH 7.4) containing 1 mM EDTA, 1 mM dithiothreitol (DTT), and 2% *(w/v)* polyvinylpyrrolidone (PVP) (all chemicals provided by Chemat, Gdańsk, Poland), according to the protocols of Homaee and Ehsanpour [14]. The homogenates were centrifuged at 13,000× *g* for 20 min at 4 °C (Centrifuge MPW-260R, MPW MED INSTRUMENTS, Warsaw, Poland). Supernatants were used to determine the total protein content and the activities of the antioxidant enzymes. The protein content was measured based on the Bradford method [29] with bovine serum albumin (BSA) as the standard. The superoxide dismutase (SOD; EC 1.15.1.1) activity was determined according to Giannopolitis and Ries [30] by measuring its ability to inhibit the photochemical reduction of nitro blue tetrazolium chloride (NBT). The guaiacol peroxidase (GPOX; EC 1.11.1.7) activity was measured using the Maehly and Chance [31] methodology as modified by Nowogórska and Patykowski [32].

The spectrophotometric analysis of the extracts was performed in the SmartSpec PlusTM spectrophotometer (BioRad, Hercules, CA, USA) at specific wavelengths (λ_max_): for chlorophyll *a* and *b* at 645 and 662 nm, for carotenoids at 470 nm, for phenolics at 765 nm, for proteins at 595 nm, for SOD at 560 nm, and for GPOX at 470. The contents of the plant pigments and the phenolic compounds were calculated per 1 g of sample fresh weight (FW). The enzymatic activity U (μmol·min^−1^) was calculated per 1 mg of protein. All of the performed biochemical analyses were repeated six times.

### 2.7. Statistical Analysis

The experiments were set up in a completely randomized design. Data were statistically verified using Statistica 13.3 (StatSoft Polska, Cracow, Poland) software. The analysis of variance (ANOVA) was performed, and means were compared with the Tukey post hoc test at the significance level of *p* ≤ 0.05. Data were presented as mean ± standard deviation (SD). For data expressed as a percentage, the Freeman–Tukey double-arcsine transformation was used. Tables with results provide numerical data, with the alphabet indicating the homogeneous groups.

## 3. Results and Discussion

**Figure 2:**
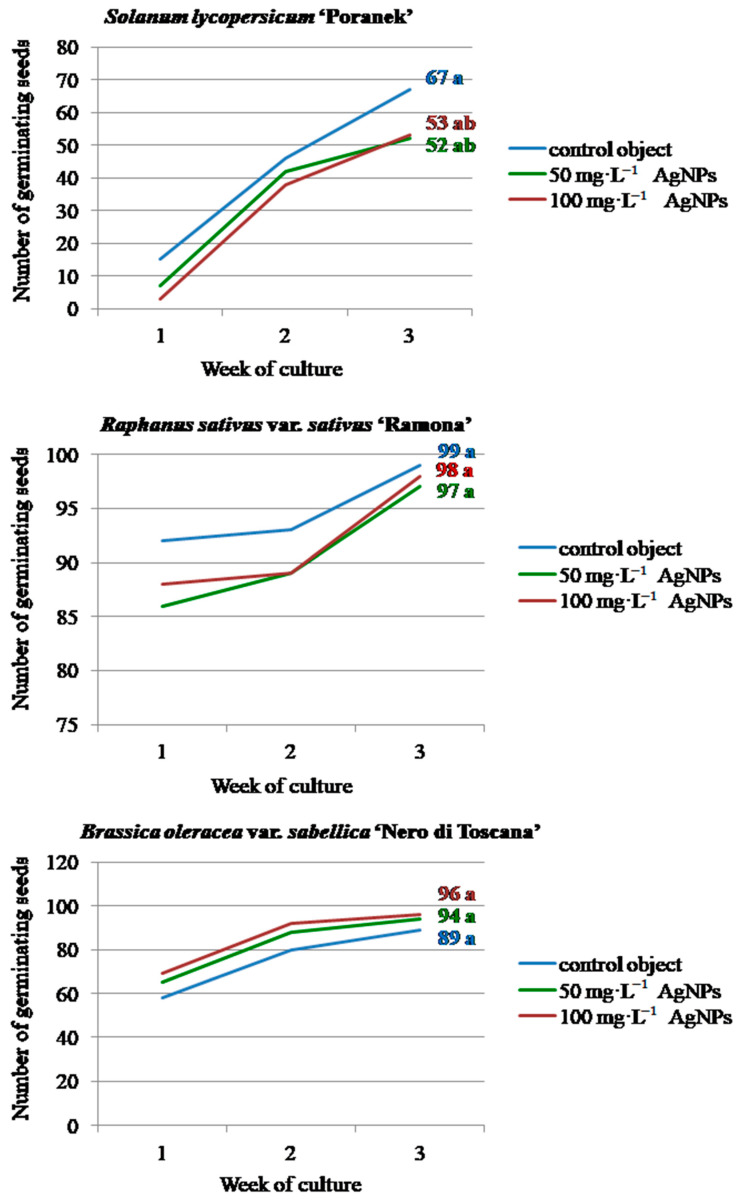
Dynamics and efficiency of in vitro seed germination in the tested vegetable plant species on the modified MS medium depending on the AgNPs treatment.

The appearance of the first germinating seeds was observed already in the first week of in vitro culture and proceeded to the third week, but with different intensities depending on the tested vegetable species (Figure 2).

During the first week, the germination of 15 control tomato seeds was observed, whereas in the experimental combinations with 50 and 100 mg·L^−1^ AgNPs, only 7 and 3 germinating seeds were reported, respectively. The highest increase in germination was found in the second week. Finally, in the third week, germination reached 67 seeds in the control object, 52 seeds in the 50 mg·L^−1^ AgNPs treatment and 53 seeds in the 100 mg·L^−1^ AgNPs treatment, without statistically significant differences. Similarly, the addition of silver nanoparticles (121.6 nm; 0.385, 0.77, 1.54, 15.4 mg·L^−1^) to the MS medium did not affect the germination rate in *Physalis peruviana* L. (Solanaceae) [6]. In the experiment performed by Mehrian et al. [33], the AgNPs-treated (50 nm; 25, 50, 75, and 100 mg·L^−1^) seeds of seven *S. lycopersicum* cultivars germinated faster, germinating within the first three days, whereas the control seeds germinated later. Interestingly, the AgNPs at concentrations of 75 and 100 mg·L^−1^ caused a significant decrease in the germination percentage in two of the tested cultivars, while no differences were observed in the germination percentage in the other five cultivars. As suggested by the authors, the NPs-accelerated seed germination in the first days after treatment may result from better water uptake by the seeds, whereas the later decrease in germination efficiency can be attributed to the accelerated breakdown of the stored nutrients and/or alternations in the permeability properties of the cell membrane resulting from AgNPs application. According to Yasur and Rani [15] and Hatami [1], water uptake during seed germination is critical because seeds are relatively dry and need a substantial amount of water to initiate cellular metabolism and growth, and positive effects of NPs on germination arise from the high capability of NPs to penetrate the seed coat and promote water uptake. In contrast, no such phenomenon occurred in this study with tomato ‘Poranek’ although due to their smaller size (20 nm), the nanoparticles used in the current research could potentially penetrate the seed coat more easily than the bigger NPs tested in the above-mentioned study. Therefore, the obtained results indicate that plant genotype is an important factor in experiments testing the influence of nanoparticles on seed germination.

As for radish, most of the seeds had already germinated in the first week of culture, with 92 seeds germinating in the control object and 86 and 88 seeds germinating after 50 mg·L^−1^ and 100 mg·L^−1^ AgNPs treatment, respectively. In the following weeks, the number of germinated seeds increased slightly (from 7 to 11). No significant difference was found between the number of germinated seeds depending on the AgNPs treatment at the end of the observation period (97, and 98, for the 50 mg·L^−1^ AgNPs and 100 mg·L^−1^ AgNPs treatments, respectively, and 99 for the control).

The vast majority of kale seeds also started to germinate during the first week of culture (65 and 69 after the 50 mg·L^−1^ AgNPs and 100 mg·L^−1^ AgNPs treatments, respectively, and 58 seeds in the control object;). The number of germinating seeds increased significantly in the second week and finally amounted to 94 and 96 after the 50 mg·L^−1^ AgNPs and 100 mg·L^−1^ AgNPs applications, respectively, and 89 in the control object. These values were not significantly different.

**Figure 3:**
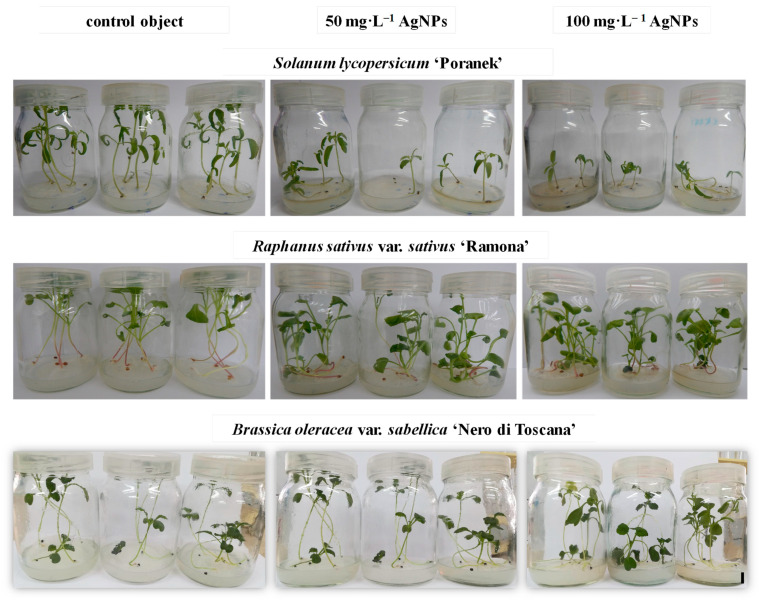
Developed seedlings of the tested vegetable plant species cultured in vitro for three weeks on the modified MS medium depending on the AgNPs treatment. Bar = 1 cm.

Silver nanoparticles that were 29 nm in size at concentrations 25–50 mg·L^−1^ did not influence the germination of *Brassica juncea* L. Czern. compared to the control. However, the efficiency of this process declined consistently with higher concentrations of AgNPs (22% decline at 400 mg·L^−1^) [34]. According to Thiruvengadam et al. [13], the germination rate in *Brassica rapa* ssp. *rapa* L. decreased when the AgNPs concentration increased although the NPs were applied at low concentrations (8 nm, 1, 5, and 10 mg·L^−1^). In *Ricinus communis* L., treatment with AgNPs (70 nm) at a very high concentration of 500–4000 mg·L^−1^ did not disturb the germination process [15]. Different results concerning the influence of silver nanoparticles on seed germination can be associated with (1) plant parameters: species and its sensitivity to AgNPs concentration and seed characteristics (size, thickness of coat) and (2) nanoparticle characteristics: type, size, concentration, surface stabilizer used, etc. During germination, the nanoparticles first have to penetrate the seed coat containing sclerenchyma (sclereids), which acts as a barrier due to its physicochemical integrity [17]. Seed coat thickness and NPs size are important factors influencing the uptake and penetration of AgNPs inside seeds. A thicker seed coat may minimize the penetration of NPs ([33]. Most likely, the low permeability of the seed coat of the tested tomato, radish, and kale genotypes could be the reason for the minimized effect of AgNPs on germination. Even though nanoparticles may not interfere with the seed germination process, as confirmed in this study with different plant species, they may affect molecular and physiological responses in developing seedlings [13]. The influence of NPs can be manifested as multidirectional changes in metabolite profiles and the biometric growth parameters that need a detailed investigation, which is addressed in this study. When the radicle emerges, the developing tissues of the root apex gain contact with NPs, which may enter the rhizodermis via apoplastic transport, endocytosis, or other carriers. Then, NPs flow within the root toward the vascular cylinder via the symplastic pathways and are translocated to other seedling parts [17]. Tomato shoots obtained from seeds treated with AgNPs at the highest concentration (100 mg·L^−1^) were characterized by the lowest length (3.64 cm), fresh weight (63.34 mg), and dry weight (3.31 mg) (Table 1, Figure 3). The highest values of these traits were reported for the control plants—7.01 cm, 211.94 mg, and 10.49 mg, respectively, whereas shoots obtained from the seeds treated with 50 mg·L^−1^ AgNPs had intermediate values. No statistically significant differences were found between the tested treatments regarding the root length (5.80–7.93 cm) and root dry weight (1.45–3.84 mg). On the other hand, the control and 100 mg·L^−1^ AgNPs-derived plants had the highest and lowest root fresh weights (48.69 mg and 10.34 mg, respectively).

As for radish, the treatment with AgNPs caused a significant decrease in the length of the shoots and a simultaneous increase in fresh and dry weight (Table 1, Figure 3). The control radish shoots were 11.69 cm high, and their fresh and dry weights were 377.05 mg and 23.59 mg, respectively. The plants obtained from seeds treated with silver nanoparticles were about 10 cm long and had fresh and dry weights of about 500 mg and 30 mg, respectively. The application of increasing concentrations of nanoparticles stimulated the elongation of the roots. Seedlings treated with 100 mg·L^−1^ AgNPs had the longest roots (12.07 cm), whereas the control plants had the shortest roots (8.87 cm). However, the root FW of the seedlings treated with 50 and 100 mg·L^−1^ AgNPs was lower (34.93 and 40.65 mg, respectively) than in the control seedlings (53.47 mg). No differences were found between the tested concentrations of silver nanoparticles in terms of the root dry weight.

In kale, the highest values for shoot fresh (196.07–245.39 mg) and dry weight (15.26–17.98 mg) were found in the plants produced from seeds treated with 50 and 100 mg·L^−1^ silver nanoparticles, but the 50 mg·L^−1^ AgNPs-derived shoots were significantly longer (11.36 cm) than those after the 100 mg·L^−1^ AgNPs-treatment (9.78 cm) and the controls (9.26 cm) (Table 1, Figure 3). The 50 mg·L^−1^ AgNPs-derived plants produced the longest roots (9.89 cm), which were also characterized by low fresh (9.22 mg) and dry (2.19 mg) weights. In contrast, the 100 mg·L^−1^ AgNPs-derived seedlings had the shortest roots (8.32 cm) but had the highest fresh (19.64 mg) and dry (2.97 mg) weights.

The low concentration of 0.385 mg·L^−1^ AgNPs promoted an increase in the fresh and dry weights of the *P. peruviana* seedlings, whereas a higher concentration (15.4 mg·L^−1^) led to a growth reduction of the shoots and roots [6]. Similarly, the shoot and root length of the *Brassica juncea* seedlings increased with concentrations of up to 200 mg·L^−1^ AgNPs and decreased with higher nanoparticle concentrations, indicating that this phenomenon is dose-dependent [34]. Treatment with AgNPs exceeding 1 mg·L^−1^ was deleterious for the growth of *B. rapa* ssp. *Rapa* seedlings. A 10% increase in the fresh weight of seedlings was recorded at 1 mg·L^−1^, while exposure to higher concentrations resulted in a gradual decrease in the fresh weight. Root length was increased at 1 mg·L^−1^ concentrations and decreased at higher concentrations. Seedlings treated with 10 mg·L^−1^ concentrations had the shortest shoots [13]. On the other hand, silver NPs applied at concentrations 500, 1000, 2000, 4000 mg·L^−1^ did not disturb the growth of *R. communis* seedlings [15]. In *S. lycopersicum*, root elongation was inhibited in the seven tested cultivars after using 25, 50, 75, and 100 mg·L^−1^ AgNPs concentrations compared to the control. As for the shoot length, a drastic decrease in the value of this parameter was usually reported, with the exception of one cultivar treated with 25 mg·L^−1^ AgNPs and another cultivar treated with 25 and 75 mg·L^−1^ concentrations. The decrease in root length was stronger than in the shoots of all of the cultivars that were studied [33]. In the tested tomato ‘Poranek’ seedlings, the values of most of the analyzed biometrical traits decreased significantly with the increasing concentrations of applied silver nanoparticles or presented decreasing tendencies. Nevertheless, the results obtained for radish ‘Ramona’ and kale ‘Nero di Toscana’ are more diverse and cannot be directly compared to the studies of other authors, thus indicating a multidirectional effect of the same nanoparticles, even when they are applied at equal concentrations on species belonging to the same botanical family.

Roots are the primary tissues through which AgNPs enter plants and are the organ that is the most responsive to nanoparticle effects. AgNPs accumulate in the initial cells of the root columella, preventing cell division and hence the growth of roots. The presence of AgNPs in plasmodesmata, precluding the transport of nutrients, could lead to the reduction of plant biomass. Moreover, AgNPs can additionally modify the absorption of metal ions important for plant development [6]. These mechanisms of NPs action could partially explain the reduction of root length found in kale reported at the concentration of 100 mg·L^−1^, the decrease of the root fresh weight and shoot parameters in tomato, and the decrease of root fresh weight and shoot length in radish. At the molecular level, nanoparticles cause the upregulation of the genes involved in cell division and carbon/nitrogen metabolism. The negative effects observed in seedling growth are probably due to chromosomal aberrations and mitotic abnormalities, leading to reduced cell division in the root meristem, hormonal imbalance, ROS overproduction, and an increased level of lipid peroxidation [17]. On the other hand, it is worth mentioning that AgNPs-treated radish and kale seedlings had higher fresh and dry weights of the shoots compared to the control plants. This genotype-specific reaction could be associated with the stimulation of cell wall lignification as a result of AgNPs application. The content and composition of lignins vary when plants are exposed to various stresses. Lignin deposition plays a crucial role in plant development and stress resistance by forming a physical barrier against stress factors. Histochemical staining proved a significant role of AgNPs treatment in inducing lignin deposition in vascular bundles in *Triticum aestivum* L. [35]. As reported by Bernard et al. [36], treatment with silver nanoparticles stimulated a more intensive lignin accumulation in the cell walls and an improved the quality of in vitro propagated *Thymus daenensis* Celak seedlings.

The stimulation of root elongation in 50 and 100 mg·L^−1^ AgNPs-treated radish seedlings might be associated with the avoidance mechanism of roots to AgNPs as a stress factor. Moreover, when nanoparticles are used at a lower concentration, the hormesis effect is often observed, i.e., a generally favorable biological response to low exposures to a toxin/stressor. This phenomenon could also explain the increase in shoot and root length in kale plants after using 50 mg·L^−1^ AgNPs [37]. Nonetheless, concerning the influence of AgNPs on the biometric parameters of seedlings, this strong genotype-specific reaction cannot be neglected.

The leaves of the control tomato seedlings produced significantly more chlorophyll *a* (1.30 mg·g^−1^ FW), chlorophyll *b* (0.54 mg·g^−1^ FW), total chlorophyll (1.84 mg·g^−1^ FW), and carotenoids (0.36 mg·g^−1^ FW) than seedlings exposed to AgNPs (Table 2). A similar phenomenon was found in kale plants depending on the silver nanoparticle treatment. On the other hand, the chlorophyll *a*/*b* ratio was higher in AgNPs-treated tomato plants (3.43 for 50 mg·L^−1^; 3.05 for 100 mg·L^−1^) than in the untreated control plants (2.45), whereas the highest and the lowest chlorophylls/carotenoids ratio was reported in the control object (5.05) and in the 50 mg·L^−1^ AgNPs-treatment plants (4.42), respectively. The kale seedlings did not differ in terms of chlorophyll *a*/*b* (2.37–2.52) and chlorophylls/carotenoids (5.19–5.60) ratios, regardless of the AgNPs treatment. In contrast, in radish, the highest content of chlorophyll *a* (1.25 mg·g^−1^ FW), chlorophyll *b* (0.50 mg·g^−1^ FW), total chlorophyll (1.75 mg·g^−1^ FW), and carotenoids (0.30 mg·g^−1^ FW) were detected in seedlings after the application of 50 mg·L^−1^ AgNPs. The highest ratio of chlorophyll *a*/*b* (2.90) and the lowest ratio of chlorophylls/carotenoids (5.60) were found in the radish control plants.

Treatment with silver nanoparticles did not affect the content of phenolic compounds in tomato (1.52–1.57 mg·g^−1^ FW) and kale (1.02–1.08 mg·g^−1^ FW) seedlings compared to the control objects—1.50 and 1.05 mg·g^−1^ FW, respectively (Table 2). In contrast, the radish seedlings from 100 mg·L^−1^ AgNPs-treatment plants had the lowest phenolics content (1.51 mg·g^−1^ FW). Varied results were obtained regarding the anthocyanins accumulation in the leaves of the tested vegetable species. In tomato, the highest content of these compounds was reported in the 100 mg·L^−1^ AgNPs-derived seedlings (0.23 mg·g^−1^ FW), while the lowest concentration was detected in the control seedlings (0.17 mg·g^−1^ FW). Conversely, the radish control seedlings produced more pigments from this group (0.30 mg·g^−1^ FW) than seedlings from the 100 mg·L^−1^ AgNPs experimental object (0.22 mg·g^−1^ FW). The exposure to silver nanoparticles at the concentrations of 50 and 100 mg·L^−1^ significantly decreased the anthocyanins synthesis in kale.

The treatment of seeds with AgNPs did not influence the total protein content in the leaves of the three-week-old tomato (26.25–26.99 mg·g^−1^ FW) and kale (13.21–13.35 mg·g^−1^ FW) seedlings (Table 2). On the other hand, the 100 mg·L^−1^ AgNPs-derived radish seedlings contained significantly less protein (12.26 mg·g^−1^ FW) than seedlings from the 50 mg·L^−1^ treatment (13.41 mg·g^−1^ FW) and the control (13.77 mg·g^−1^ FW). In each species, none of the tested experimental objects differed in terms of SOD activity. In contrast, in tomato, the application of 100 mg·L^−1^ AgNPs resulted in an almost two-fold higher GPOX activity (10.85 U) than in the control seedlings (5.54 U). Radish seeds exposed to 100 mg·L^−1^ AgNPs produced seedlings with lower guaiacol peroxidase activity (2.18 U) than the control seedlings (5.16 U) and the seedlings in the 50 mg·L^−1^ treatment (4.12 U). As for kale, a significant increase in GPOX activity was reported in the seedlings treated with silver nanoparticles at the concentration of 50 mg·L^−1^ (3.09 U) and 100 mg·L^−1^ (4.11 U) compared to the nontreated control seedlings (1.28 U).

Seed priming with AgNPs significantly affected photosynthetic parameters such as the chlorophyll and carotenoid content, photosynthetic rate, stomatal conductance, and transpiration rate in *P. glaucum*, increasing the plant length and yield [7]. An increase in the chlorophyll content in 7-day-old *B. juncea* was observed for the 25, 50, and 100 mg·L^−1^ AgNPs-treatments [34]. Interestingly, AgNPs at 1 mg·L^−1^ did not affect the total chlorophyll content, whereas higher concentrations (5 and 10 mg·L^−1^) resulted in a significantly lower total chlorophyll content in 2-week-old *B. rapa* ssp. *rapa* seedlings [13]. The treatment with 50 mg·L^−1^ AgNPs increased the chlorophyll and carotenoid content in the radish ‘Ramona’ seedlings, whereas the 100 mg·L^−1^-treatment decreased the values of these parameters. Similarly, all AgNPs-treated tomato ‘Poranek’ and kale ‘Nero di Toscana’ seedlings were characterized by lower chlorophyll and carotenoid contents. The reduction in the chlorophyll content was significant at the higher AgNPs concentrations (1.54 and 15.4 mg·L^−1^) in the *P.s peruviana* seedlings [6]. The loss of chlorophyll negatively affects photosynthesis and causes a surplus of electrons to combine with molecular oxygen, forming injurious ROS that may irreversibly damage the structure of chloroplasts [13] and, finally, may deteriorate seedling biometric parameters, causing growth inhibition.

In the conducted experiments, the anthocyanin content was significantly lower in all of the AgNPs-treated kale seedlings and in the 100 mg·L^−1^-treated radish seedlings, whereas the 100 mg·L^−1^-treated tomato seedlings had a higher content of these compounds. Anthocyanin content was significantly increased with higher concentrations of AgNPs in *B. rapa* ssp. *rapa* seedlings. The accumulation of anthocyanins resulting from AgNPs exposure might be due to higher oxidative stress caused by the AgNPs. Moreover, the 5 and 10 mg·L^−1^ AgNPs induced the expression of genes related to phenolics biosynthesis and down-regulated the carotenoids gene expression [13]. The polyphenol content in the *R. communis* shoots increased at 500 and 1000 mg·L^−1^ treatments and decreased gradually at the 2000 and 4000 mg·L^−1^ AgNPs treatments [15]. Therefore, it can be concluded that the differences in the level of the discussed compounds vary depending on the specific AgNPs treatment and genotype tested. Moreover, different results can be obtained depending on the age of the analyzed plant material or the time between NPs-treatment and biochemical analysis performance.

With respect to the influence of silver nanoparticles on the activity of enzymes associated with oxidative stress, no differences were reported in the SOD and APX activities in *P. peruviana* [6]. On the other hand, peroxidase, catalase, and superoxide dismutase activity were enhanced in the 500–4000 mg·L^−1^ AgNPs treatments in *R. communis* [15]. As for *P. glaucum*, silver nanoparticles improved superoxide dismutase and catalase activity but decreased peroxide activity [7]. In *B. juncea*, GPOX activity increased continuously with an increasing concentration of AgNPs, reaching the maximum at 400 mg·L^−1^. No differences were reported in the activity of CAT in the AgNPs concentration range of 25–100 mg·L^−1^. The activity of this enzyme was significantly higher at 200 and 400 mg·L^−1^. The lowest APX activity was reported at 50 mg·L^−1^, whereas the highest activity was observed at 200 and 400 mg·L^−1^ [34]. The SOD, CAT, APX, and glutathione reductase (GR) activity were influenced by AgNPs treatment (20 nm; 2, 10, 20 mg·L^−1^) in 4-week-old *Solanum tuberosum* L. plants. Interestingly, the activities of these enzymes were found at the maximum level at 10 mg·L^−1^ AgNPs but declined at 20 mg·L^−1^ [14]. How can the varied results concerning the SOD and GPOX activity in the tested tomato, radish, and kale seedlings be explained? The long duration of the in vitro culture period (or the period between NPs treatment and the determination of enzyme activity) as well as exposure to light irradiation might be associated with changes in nanoparticle size through agglomeration and decreases in their toxicity, consequently changing the activity levels of enzymatic antioxidants over time [6]. Interestingly, both the elevation and reduction of antioxidant enzyme activities can be related to oxidative stress. Moreover, the inactivation of SOD under severe oxidative stress is also possible [14]. The influence of the genotype should also not be neglected.

Interestingly, contrary results regarding the germination efficiency, growth, and biochemical activity of seedlings, depending on the species tested and type of nanoparticles used, were reported by other authors, e.g., in *P. glaucum* [38] and *Zea mays* L. [39] treated with gold nanoparticles; in *Lactuca sativa* L. treated with silica, palladium, gold, and copper nanoparticles [40]; in *Triticum aestivum* L. treated with copper and zinc oxide [41]; or in *Capsicum annuum* L. treated with zinc oxide nanoparticles [42]. These reports correspond with the results of the present experiment and bring attention to the multifaced characteristics of interactions between plants and nanoparticles.

## 4. Conclusions

The further development of nanotechnology is inevitable, and the emission of various ENMs to the environment will dynamically increase. The spectrum of possible interactions between nanomaterials and living organisms, including plants, is very diverse. Contradictions in plant responses to ENMs depend on several factors, i.e., plant genotype, ENMs type and properties, method and time of ENMs treatment, or interactions with different environmental factors. Each subsequent experiment broadening the scope of knowledge on the influence of nanomaterials on plants seems to be fully reasonable. The results presented in this paper indicate that silver nanoparticles of the same size, at the same concentration, and applied with a uniform methodology affect different biometric and biochemical responses depending on whether the tested plant species belongs to the same or different botanical family. Future studies should aim to test different types and concentrations of ENMs to evaluate their possible utility in the micropropagation of vegetable plant species and to verify the genetic and phenotype stability of the treated plants for breeding purposes.

## Figures and Tables

**Table 1:** Influence of AgNPs application in the tested vegetable plant species on the biometric parameters of the seedlings after three weeks of in vitro culture on the modified MS medium.

Concentration of AgNPs	Shoot Length (cm)	Shoot Fresh Weight (mg)	Shoot Dry Weight (mg)	Root Length (cm)	Root Fresh Weight (mg)	Root Dry Weight (mg)
	*Solanum lycopersicum* ‘Poranek’					
control object	7.01 ± 3.14 a	211.94 ± 161.39 a	10.49 ± 2.18 a	7.93 ± 6.48 a	48.69 ± 56.94 a	3.84 ± 1.33 a
50 mg·L^−1^	5.47 ± 1.21 ab	143.52 ± 39.08 ab	7.94 ± 3.46 ab	6.62 ± 2.35 a	25.28 ± 7.79 ab	2.64 ± 1.59 a
100 mg·L^−1^	3.64 ± 0.89 b	63.34 ± 46.15 b	3.31 ± 2.10 b	5.80 ± 2.54 a	10.34 ± 7.30 b	1.45 ± 0.59 a
	*Raphanus sativus* var. *sativus* ‘Ramona’					
control object	11.69 ± 2.30 a	377.05 ± 109.06 b	23.59 ± 2.33 b	8.87 ± 5.62 b	53.47 ± 27.29 a	3.63 ± 0.89 a
50 mg·L^−1^	9.97 ± 3.65 b	496.03 ± 249.87 a	29.32 ± 3.97 a	10.79± 5.75ab	34.93 ± 24.81 b	3.82 ± 1.08 a
100 mg·L^−1^	9.98 ± 2.96 b	515.06 ± 206.82 a	30.14 ± 3.40 a	12.07 ± 3.72 a	40.65 ± 24.20 b	3.65 ± 0.71 a
	*Brassica oleracea* var. *sabellica* ‘Nero di Toscana’					
control object	9.26 ± 2.69 b	130.45 ± 41.20 b	10.30 ± 0.84 b	8.49 ± 1.86 ab	15.13 ± 7.43 ab	2.00 ± 0.33 b
50 mg·L^−1^	11.36 ± 2.53 a	196.07 ± 107.55 ab	15.26 ± 3.12 a	9.89 ± 2.80 a	9.22 ± 8.50 b	2.19 ± 0.43 b
100 mg·L^−1^	9.78 ± 3.26 b	245.39 ± 126.41 a	17.98 ± 0.66 a	8.32 ± 2.77 b	19.64 ± 14.66 a	2.97 ± 0.48a ^1^

^1^ Values represent means ± standard deviation. Means in columns for each tested vegetable plant species followed by the same letter do not differ significantly at *p* ≤ 0.05 (Tukey’s test).

**Table 2:** Influence of AgNPs application in the tested vegetable plant species on the biochemical parameters of seedlings after three weeks of in vitro culture on the modified MS medium.

Concentration of AgNPs	Chlorophyll *a* Content (mg·g^−1^ FW)	Chlorophyll *b* Content (mg·g^−1^ FW)	Chlorophyll *a/b* Ratio	Chlorophylls (*a + b*) Content (mg·g^−1^ FW)	Carotenoids Content (mg·g^−1^ FW)	Chlorophylls/ Carotenoids Ratio	Anthocyanins Content (mg·g^−1^ FW)	Phenolic Compounds Content (mg·g^−1^ FW)	Total Protein Content (mg·g^−1^ FW)	SOD Activity (U)	GPOX Activity (U)
*Solanum lycopersicum* ‘Poranek’											
control object	1.30 ± 0.25 a	0.54 ± 0.13 a	2.45 ± 0.14 b	1.84 ± 0.38 a	0.36 ± 0.07 a	5.05 ± 0.10 a	0.17 ± 0.03 b	1.50 ± 0.09 a	26.64 ± 0.68 a	2.48 ± 0.75 a	5.54 ± 0.66 b
50 mg·L^−1^	0.77 ± 0.10 b	0.23 ± 0.04 b	3.43 ± 0.25 a	1.00 ± 0.14 b	0.23 ± 0.02 b	4.42 ± 0.20 b	0.19 ± 0.02 ab	1.52 ± 0.11 a	26.25 ± 1.57 a	2.20 ± 1.06 a	6.79 ± 2.16 b
100 mg·L^−1^	0.83 ± 0.17 b	0.28 ± 0.09 b	3.05 ± 0.51 a	1.12 ± 0.26 b	0.24 ± 0.05 b	4.69 ± 0.36 ab	0.23 ± 0.03 a	1.57 ± 0.16 a	26.99 ± 1.16 a	4.11 ± 0.79 a	10.85 ± 0.78 a
*Raphanus sativus* var. *sativus* ‘Ramona’											
control object	1.10 ± 0.05 ab	0.38 ± 0.02 b	2.90 ± 0.26 a	1.48 ± 0.05 ab	0.27 ± 0.02 ab	5.60 ± 0.34 b	0.30 ± 0.05 a	1.65 ± 0.08 ab	13.77 ± 0.25 a	7.18 ± 2.24 a	5.16 ± 1.03 a
50 mg·L^−1^	1.25 ± 0.20 a	0.50 ± 0.10 a	2.52 ± 0.17 b	1.75 ± 0.30 a	0.30 ± 0.05 a	5.92 ± 0.17 ab	0.29 ± 0.06 ab	1.82 ± 0.10 a	13.41 ± 0.31 a	6.56 ± 1.71 a	4.12 ± 0.74 a
100 mg·L^−1^	1.02 ± 0.04 b	0.39 ± 0.02 b	2.61 ± 0.09 b	1.41 ± 0.06 b	0.23 ± 0.01 b	6.18 ± 0.13 a	0.22 ± 0.05 b	1.51 ± 0.19 b	12.16 ± 0.51 b	8.36 ± 0.98 a	2.18± 0.94b
*Brassica oleracea* var. *sabellica* ‘Nero di Toscana’											
control object	1.76 ± 0.11 a	0.70 ± 0.04 a	2.52 ± 0.05 a	2.45 ± 0.14 a	0.48 ± 0.03 a	5.19 ± 0.10 a	0.13 ± 0.03 a	1.05 ± 0.39 a	13.21 ± 0.64 a	2.63 ± 0.11 a	1.28 ± 0.51 b
50 mg·L^−1^	1.41 ± 0.10 b	0.58 ± 0.06 b	2.47 ± 0.27 a	1.99 ± 0.14 b	0.36 ± 0.03 b	5.60 ± 0.48 a	0.09 ± 0.02 c	1.02 ± 0.04 a	13.35 ± 0.13 a	2.64 ± 0.13 a	3.09 ± 1.87 ab
100 mg·L^−1^	1.54 ± 0.17 b	0.65 ± 0.09 b	2.37 ± 0.18 a	2.19 ± 0.25 ab	0.40 ± 0.05 b	5.49 ± 0.27 a	0.11 ± 0.03 b	1.08 ± 0.01 a	13.23 ± 0.20 a	2.52 ± 0.18 a	4.11 ± 1.13 a ^1^

^1^ Values represent means ± standard deviation. Means in columns for each tested vegetable plant species followed by the same letter do not differ significantly at *p* ≤ 0.05 (Tukey’s test).

## Data Availability

The data presented in this study are available upon request.
